# Simulation-based summative assessment in healthcare: an overview of key principles for practice

**DOI:** 10.1186/s41077-022-00238-9

**Published:** 2022-12-28

**Authors:** Clément Buléon, Laurent Mattatia, Rebecca D. Minehart, Jenny W. Rudolph, Fernande J. Lois, Erwan Guillouet, Anne-Laure Philippon, Olivier Brissaud, Antoine Lefevre-Scelles, Dan Benhamou, François Lecomte, the SoFraSimS Assessment with simulation group, Anne Bellot, Isabelle Crublé, Guillaume Philippot, Thierry Vanderlinden, Sébastien Batrancourt, Claire Boithias-Guerot, Jean Bréaud, Philine de Vries, Louis Sibert, Thierry Sécheresse, Virginie Boulant, Louis Delamarre, Laurent Grillet, Marianne Jund, Christophe Mathurin, Jacques Berthod, Blaise Debien, Olivier Gacia, Guillaume Der Sahakian, Sylvain Boet, Denis Oriot, Jean-Michel Chabot

**Affiliations:** 1 Department of Anesthesiology, Intensive Care and Perioperative Medicine, Caen Normandy University Hospital, 6th Floor, Caen, France; 2 Medical School, University of Caen Normandy, Caen, France; 3 Center for Medical Simulation, Boston, MA USA; 4 Department of Anesthesiology, Intensive Care and Perioperative Medicine, Nîmes University Hospital, Nîmes, France; 5 Department of Anesthesia, Critical Care and Pain Medicine, Massachusetts General Hospital, Boston, MA USA; 6 Harvard Medical School, Boston, MA USA; 7 Department of Anesthesiology, Intensive Care and Perioperative Medicine, Liège University Hospital, Liège, Belgique; 8 Department of Emergency Medicine, Pitié Salpêtrière University Hospital, APHP, Paris, France; 9 Department of Pediatric Intensive Care, Pellegrin University Hospital, Bordeaux, France; 10 Department of Emergency Medicine, Rouen University Hospital, Rouen, France; 11 Department of Anesthesiology, Intensive Care and Perioperative Medicine, Kremlin Bicêtre University Hospital, APHP, Paris, France; 12 Department of Emergency Medicine, Cochin University Hospital, APHP, Paris, France

**Keywords:** Medical education, Summative, Assessment, Simulation, Education, Competency-based education

## Abstract

**Background:**

Healthcare curricula need summative assessments relevant to and representative of clinical situations to best select and train learners. Simulation provides multiple benefits with a growing literature base proving its utility for training in a formative context. Advancing to the next step, “the use of simulation for summative assessment” requires rigorous and evidence-based development because any summative assessment is high stakes for participants, trainers, and programs. The first step of this process is to identify the baseline from which we can start.

**Methods:**

First, using a modified nominal group technique, a task force of 34 panelists defined topics to clarify the why, how, what, when, and who for using simulation-based summative assessment (SBSA). Second, each topic was explored by a group of panelists based on state-of-the-art literature reviews technique with a snowball method to identify further references. Our goal was to identify current knowledge and potential recommendations for future directions. Results were cross-checked among groups and reviewed by an independent expert committee.

**Results:**

Seven topics were selected by the task force: “What can be assessed in simulation?”, “Assessment tools for SBSA”, “Consequences of undergoing the SBSA process”, “Scenarios for SBSA”, “Debriefing, video, and research for SBSA”, “Trainers for SBSA”, and “Implementation of SBSA in healthcare”. Together, these seven explorations provide an overview of what is known and can be done with relative certainty, and what is unknown and probably needs further investigation. Based on this work, we highlighted the trustworthiness of different summative assessment-related conclusions, the remaining important problems and questions, and their consequences for participants and institutions of how SBSA is conducted.

**Conclusion:**

Our results identified among the seven topics one area with robust evidence in the literature (“What can be assessed in simulation?”), three areas with evidence that require guidance by expert opinion (“Assessment tools for SBSA”, “Scenarios for SBSA”, “Implementation of SBSA in healthcare”), and three areas with weak or emerging evidence (“Consequences of undergoing the SBSA process”, “Debriefing for SBSA”, “Trainers for SBSA”). Using SBSA holds much promise, with increasing demand for this application. Due to the important stakes involved, it must be rigorously conducted and supervised. Guidelines for good practice should be formalized to help with conduct and implementation. We believe this baseline can direct future investigation and the development of guidelines.

## Background

There is a critical need for summative assessment in healthcare education [1]. Summative assessment is high stakes, both for graduation certification and for recertification in continuing medical education [2–5]. Knowing the consequences, the decision to validate or not validate the competencies must be reliable, based on rigorous processes, and supported by data [6]. Current methods of summative assessment such as written or oral exams are imperfect and need to be improved to better benefit programs, learners, and ultimately patients [7]. A good summative assessment should sufficiently reflect clinical practice to provide a meaningful assessment of competencies [1, 8]. While some could argue that oral exams are a form of verbal simulation, hands-on simulation can be seen as a solution to complement current summative assessments and enhance their accuracy by bringing these tools closer to assessing the necessary competencies [1, 2].

Simulation is now well established in the healthcare curriculum as part of a modern, comprehensive approach to medical education (e.g., competency-based medical education) [9–11]. Rich in various modalities, simulation provides training in a wide range of technical and non-technical skills across all disciplines. Simulation adds value to the educational training process particularly with feedback and formative assessment [9]. With the widespread use of simulation in the formative setting, the next logical step is using simulation for summative assessment.

The shift from formative to summative assessment using simulation in healthcare must be thoughtful, evidence-based, and rigorous. Program directors and educators may find it challenging to move from formative to summative use of simulation. There are currently limited experiences (e.g., OSCE [12, 13]) but not established guidelines on how to proceed. The evidence needed for the feasibility, the validity, and the definition of the requirement for simulation-based summative assessment (SBSA) in healthcare education has not yet been formally gathered. With this evidence, we can hope to build a rigorous and fair pathway to SBSA.

**Fig. 1:**
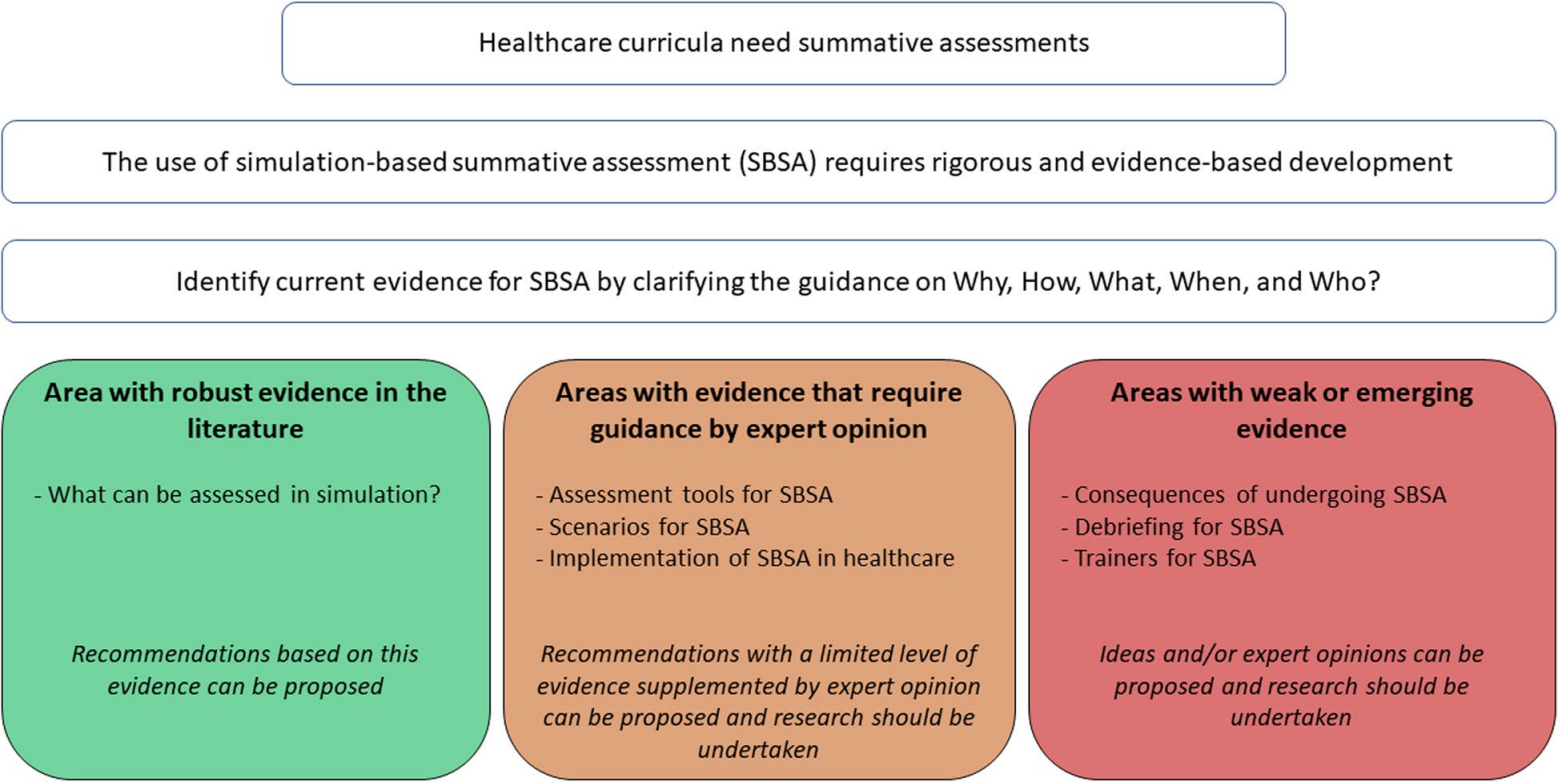
Study question and topic level of evidence

The purpose of this work is to review current knowledge for SBSA by clarifying the guidance on why, how, what, when, and who. We aim at identifying areas (i) with robust evidence in the literature, (ii) with evidence that requires guidance by expert opinion, and (iii) with weak or emerging evidence. This may serve as a basis for future research and guideline development for the safe and effective use of SBSA (Fig. 1).

## Methods

First, we performed a modified Nominal Group Technique (NGT) to define the further questions to be explored in order to have the most comprehensive understanding of SBSA. We followed recommendations on NGT for conducting and reporting this research [14]. Second, we conducted state-of-the-art literature reviews to assess the current knowledge on the questions/topics identified by the modified NGT. This work did not require Institutional Review Board involvement.

### Context

A discussion on the use of SBSA was led by executive committee members of the *Société Francophone de Simulation en Santé* (SoFraSimS) in a plenary session and involved congress participants in May 2018 at the SoFraSimS annual meeting in Strasbourg, France. Key points addressed during this meeting were the growing interest in using SBSA, its informal uses, and its inclusion in some formal healthcare curricula. The discussion identified that these important topics lacked current guidelines. To reduce knowledge gaps, the SoFraSimS executive committee assigned one of its members (FL, one of the authors) to lead and act as a NGT facilitator for a task force on SBSA. The task force’s mission was to map the current landscape of SBSA, the current knowledge and gaps; and potentially to identify experts’ recommendations.

### Task force characteristics

The task force panelists were recruited among volunteer simulation healthcare trainers in French-speaking countries after a call for application by SoFraSimS in May 2019. Recruiting criteria were a minimum of 5 years of experience in simulation and a direct involvement in simulation programs development or currently running. There were 34 (12 women and 22 men) from 3 countries (Belgium, France, Switzerland) included. Twenty-three were physicians and 11 were nurses, while 12 total had academic positions. All were experienced trainers in simulation for more than 7 years and were involved or responsible for initial training or continuing education programs with simulation. The task force leader (FL) was responsible for recruiting panelists, organizing, and coordinating the modified NGT, synthesizing responses, and writing the final report. A facilitator (CB) assisted the task force leader with the modified NGT, the synthesis of responses, and the writing of the final report. Both NGT facilitators (FL and CB) had more than 14 years of experience in simulation, had experience in research in simulation, and were responsive to simulation programs development and running.

### First part: initial question and modified nominal group technique (NGT)

**Fig. 2:**
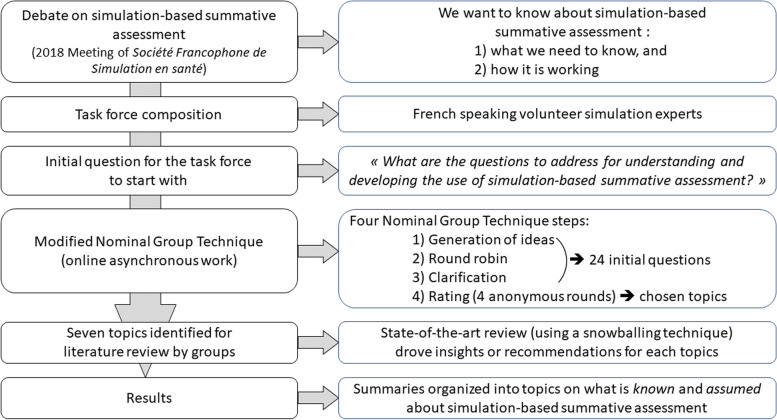
Study flowchart

To answer the challenging question of “What do we need to know for a safe and effective SBSA practice?”, following the French *Haute Autorité de Santé* guidelines [15], we applied a modified nominal group technique (NGT) approach [16] between September and October 2019. The goal of our modified NGT was to define the further questions to be explored to have the most comprehensive understanding of the current SBSA (Fig. 2). The modifications to NGT included interactions that were not in-person and were asynchronous for some. Those modifications were introduced as a result of the geographic dispersion of the panelists across multiple countries and the context of the COVID-19 pandemic.

The first two steps of the NGT (generation of ideas and round robin) facilitated by the task force leader (FL) were conducted online simultaneously and asynchronously via email exchanges and online surveys over a 6-week period. For the initiation of the first step (generation of ideas), the task force leader (FL) sent an initial non-exhaustive literature review of 95 articles and proposed the initial following items for reflection: definition of assessment, educational principles of simulation, place of summative assessment and its implementation, assessment of technical and non-technical skills in initial training, continuing education, and interprofessional training. The task force leader (FL) asked the panelists to formulate topics or questions to propose for exploration in Part 2 based on their knowledge and the literature provided Panelists independently elaborated proposals and sent them back to the task force leader (FL) who regularly synthesized them and sent the status of the questions/topics to the whole task force while preserving the anonymity of the contributors and asking them to check the accuracy of the synthesized elements (second step, as a “round robin”).

The third step of the NGT (clarification) was carried out during a 2-h video conference session. All panelists were able to discuss the proposed ideas, group the ideas into topics, and make the necessary clarifications. As a result of this step, 24 preliminary questions were defined for the fourth step (Supplemental Digital Content 1).

The fourth step of the NGT (vote) consisted of four distinct asynchronous and anonymous online vote rounds that led to a final set of topics with related sub-questions (Supplemental Digital content 2). Panelists were asked to vote to regroup, separate, keep, or discard questions/topics. All vote rounds followed similar validation rules. We [NGT facilitators (FL and CB)] kept items (either questions or topics) with more than 70% approval ratings by panelists. We reworded and resubmitted in the next round all items with 30–70% approval. We discarded items with less than 30% approval. The task force discussed discrepancies and achieved final ratings with a complete agreement for all items. For each round, we sent reminders to reach a minimum participation rate of 80% of the panelists. Then, we split the task force into 7 groups, one for each of the 7 topics defined at the end of the vote (step 4).

### Second part: literature review

From November 2019 to October 2020, the groups each identified existing literature containing the current knowledge, and potential recommendations on the topic they were to address. This identification was done based on a non-systematic review of the existing literature. To identify existing literature, the groups conducted state-of-the-art reviews [17] and expanded their reviews with a snowballing literature review technique [18] based on the articles’ references. The selected literature search performed by each group was inserted into the task force's common library on SBSA in healthcare as it was conducted.

For references, we searched electronic databases (MEDLINE), gray literature databases (including digital theses), simulation societies and centers’ websites, generic web searches (e.g., Google Scholar), and reference lists from articles. We selected publications related to simulation in healthcare with keywords “summative assessment,” “summative evaluation,” and also specific keywords related to topics. The search was iterative to seek all available data until saturation was achieved. Ninety-five references were initially provided to the task force by the NGT facilitator leader (FL). At the end of the work, the task force common library contained a total of 261 references.

### Techniques to enhance trustworthiness from primary reports to the final report

The groups’ primary reports were reviewed and critiqued by other groups. After group cross-reviewing, primary reports were compiled by NGT facilitators (FL and CB) in a single report. This report, responding to the 7 topics, was drafted in December 2020 and submitted as a single report to an external review committee composed of 4 senior experts in education, training, and research from 3 countries (Belgium, Canada, France) with at least 15 years of experience in simulation. NGT facilitators (FL and CB) responded directly to reviewers when possible and sought assistance from the groups when necessary. The final version of the report was approved by the SoFraSimS executive committee in January 2021.

## Results

### First part: modified nominal group technique (NGT)

The first two steps of the NGT by their nature (generation of ideas and “round robin”) did not provide results. The third step (clarification phase), identified 24 preliminary questions (Supplemental digital content 1) to be submitted to the fourth step (vote). The 4 rounds of voting (step 4) resulted in 7 topics with sub-questions (Supplemental Digital content 2): (1) “What can be assessed in simulation?” (2) “Assessment tools for SBSA,” (3) “Consequences of undergoing the SBSA process,” (4) “Simulation scenarios for SBSA,” (5) “Debriefing, video, research and SBSA strategies,” (6) Trainers for SBSA,” (7) “Implementation of SBSA in healthcare”. These 7 topics and their sub-questions were the starting point for the state-of-the-art literature reviews of each group for the second part.

### Second part: literature review

For each of the 7 topics, the groups highlighted what appears to be validated in the literature, the remaining important problems and questions, and their consequences for participants and institutions of how SBSA is conducted. Results in this section present the major ideas and principles from the literature review, including their nuances where necessary.

#### What can be assessed in simulation?

Healthcare faculty and institutions must ensure that each graduate is practice ready. Readiness to practice implies mastering certain competencies, which is dependent on learning them appropriately. The competency approach involves explicit definitions of the acquired core competencies necessary to be a “good professional.” Professional competency could be defined as the ability of a professional to use judgment, knowledge, skills, and attitudes associated with their profession to solve complex problems [19–21]. Competency is a complex “knowing how to act” based on the effective mobilization and combination of a variety of internal and external resources in a range of situations [19]. Competency is not directly observable; it is the performance in a situation that can be observed [19]. Performance can vary depending on human factors such as stress, fatigue, etc.… During simulation, competencies can be assessed by observing “key” actions using assessment tools [22]. Simulation’s limitations must consider when defining the assessable competencies. Not all simulation methods are equivalent to assessing specific competencies [22].

Most healthcare competencies can be assessed with simulation, throughout at curriculum, if certain conditions are met. First, the competency being assessed summatively must have already been assessed formatively with simulation [23, 24]. Second, validated assessment tools must be available to conduct this summative assessment [25, 26]. These tools must be reliable, objective, reproducible, acceptable, and practical [27–30]. The small number of currently validated tools limits the use of simulation for competency certification [31]. Third, it is not necessary or desirable to certify all competencies [32]. The situations chosen must be sufficiently frequent in the student’s future professional practice (or potentially impactful for the patient) and must be hard or impossible to assess and validate in other circumstances (e.g., clinical internships) [2]. Fourth, simulation can be used for certification throughout the curriculum [33–35]. Finally, limitations for the use of simulation throughout the curriculum may be a lack of logistical resources [36]. Based on our findings in the literature, we have summarized in Table 1 the educational consideration when implementing a SBSA.

**Table 1:** Considerations for implementing a summative assessment with simulation

Considerations	Elements	Items	Example adapted to cardiopulmonary resuscitation (CPR) for an emergency physician
**Competency to be assessed**	**Clear definition of competency**	*Know how* to act in a professional situation Identify *internal resources*: knowledge, skills, behavior, and reasoning Identify *external resources*: equipment, written or electronic resources), colleagues, and so on to mobilize	The practitioner is able to handle an in-hospital cardiac arrest (CA) ACLS algorithm, airway management, leadership, management according to the type of CA (e.g., asystole, pulseless electrical activity, ventricular fibrillation) e.g., defibrillator, cognitive aids (a chart, a checklist, …), ECMO team, …
	**Number of competencies**	Consider the possibility of assessing one or more competencies simultaneously	In-hospital CA alone, or CA in adult patient and/or in specific conditions (e.g., child, pregnant, …)
	**Measurements**	Consider measuring performance in representative and diverse situations	CA in a young polytrauma patient, in an elderly diabetic patient, in a pregnant woman or in a child out-of-hospital
**Assessment**	**Context authenticity**	Complex problems Adapt the complexity to the training level Ensure context relevance to future or current professional practice Interprofessional situations (vs uniprofessional)	e.g., CA due to hyperkaliemia in a patient with renal failure Complexity may be tuned for an expert with patient's chronic use of beta-blockers CA occurs in an ambulance or in an emergency room or in OR or in ICU Prefer a situation where the learner is not alone such as a member of an emergency team and not as a first responder in the street
	**Standardization**	Tasks and requirements known before by the participants Direct observation associated with a phase of student interaction (questioning) Rate with a checklist or a rubric	Send to the learner the assessment template prior to the assessment The simulation is followed by a debriefing (feedback)
	**Correction criteria**	Multiple sources and/or iteration (e.g., repeated performances of the same scenario) Clear and specific objectives Adjusted to the assessed knowledge or to the simulation Integration of self-assessment Consider only important errors Strategies (cognitive and metacognitive) assessed during the interaction phase Prior consensus on rating and definition regarding expected level of development	e.g., time from the start of VF to the first external electric shock and/or compliance with ACLS steps and/or quality of external cardiac massage (visual and/or via sensors) Only items that have been previously decided are assessed (see above) It is not possible to assess the use of the defibrillator if the situation is pulseless electrical activity 6 instead of 5 min between 2 doses of adrenaline (minor error) versus no recognition of a shockable rhythm (major error) Ask questions during feedback phase: “Can you remind me of the administration schedule for epinephrine in CA?” (cognition). “I have observed that you administered it every minute, but as you have just said and as I think it is every 3 to 5 min, could you explain why in the situation you administered it every minute?” (metacognition) Identify minor and major errors together (all instructors involved in the assessment of this competency). Define the number of acceptable minor and/or major errors to validate the acquisition or not of the competency at this level of development
**Scenarios**	**Development**	Developing scenarios only after defining the skills and or competences to be assessed Ensuring the scenario reflects professional reality Incorporating the targeted skills into a scenario representing professional practice, rather than a task trainer, for example	e.g., if we want to evaluate the use of the defibrillator, we need to construct a scenario where the patient has VF or VT e.g., use a hyperkalemia CA scenario after a burial extraction but not when releasing a tourniquet after a knee replacement for an emergency physician Prefer to use a scenario with a clinical history of CA to assess CPR skills rather than performing CPR in a skill station
	**Multiple skills**	Several stations with short scenarios (e.g., 5–6 min) each are preferable to long scenarios (e.g., > 20 min) Critical situation	Ensure that all steps can be assessed. E.g., the use of ECMO is reserved for refractory CA and cannot be considered if the scenario lasts for 5 min and begins with the recognition of the arrest. In this case, a scenario with a CA that has already been under management for 15 min should be used
	**Test prior to use**	Validity, reliability, reproducibility	The scenarios used should be pre-tested by the teaching team including using the assessment forms
	**Simulators (High and low-Technology)**	Use and difficulty level validated	e.g., if intubation is expected during the scenario, the chosen manikin should allow it
**Assessment test**	**standardization** **(Fairness)**	Facilitator's role and intervention specified in advance Only one candidate per station	What can the facilitator do? E.g., can he/she guide on 4H-4 T if the learner does not think about it?
	**Practical conditions**	Minimum number of scenarios (8 to 15) [157] Incentive to verbalize after action (Reasoning, what is done or not done)	Scenarios in different circumstances (in and out-of-hospital), different causes (4H-4 T), different ages (child to elderly adult) To be recalled in the pre-briefing
	**Raters**	At least, two raters Ideally, a rater should be involved in the formative assessment program	e.g., clinical supervisor, ACLS instructor, simulation instructor who has supervised the learner during the formative sessions, …

#### Assessment tools for simulation-based summative assessment

One of the challenges of assessing competency lies in the quality of the measurement tools [31]. A tool that allows the raters to collect data must also allow them to give meaning to their assessment, while securing that it is really measuring what it aims to [25, 37]. A tool must be valid and, capable of measuring the assessed competency with fidelity and, reliability while providing reproducible data [38]. Since a competency is not directly measurable, it will be analyzed on the basis of learning expectations, the most “concrete” and observable form of a competency [19]. Several authors have described definitions of the concept of validity and the steps to achieve it [38–41]. Despite different validation approaches, the objectives are similar: to ensure that the tool is valid, the scoring items reflect the assessed competency, and the contents are appropriated for the targeted learners and raters [20, 39, 42, 43]. A tool should have psychometric characteristics that allow users to be confident of its reproducibility, discriminatory nature, reliability, and external consistency [44]. A way to ensure that a tool has acceptable validity is to compare it to existing and validated tools that assess the same skills for the same learners. Finally, it is important to consider the consequences of the test to determine whether it best discriminates competent students from others [38, 43].

Like a diagnostic score, a relevant assessment tool must be specific [30, 39, 41]. It is not good or bad, but valid through a rigorous validation process [39, 41, 42]. This validation process determines whether the tool measures what it is supposed to measure and whether this measurement is reproducible at different times (test–retest) or with 2 observers simultaneously. It also determines if the tool results are correlated with another measure of the same ability or competency and if the consequences of the tool results are related to the learners’ actual competency [38].

Following Messick’s framework, which aimed to gather different sources of validity in one global concept (unified validity), Downing describes five sources of validity, which must be assessed with the validation process [38, 45, 46]. Table 2 presents an illustration of the development used in SBSA according to the unified validity framework for a technical task [38, 45, 46]. An alternative framework using three sources of validity for teamwork’s non-technical skills are presented in Table 3.

**Table 2:** Example of the development of a tool to assess technical skill achievement in a simulated situation, based on work by Oriot et al., Downing, and Messick’s framework [38, 46, 47]

Source of validity	Method	Judgment criteria	Results
content	1. Description of the checklist development by 2 experts 2. Review by 2 outside experts 3. Definitive Checklist	Relevance of items Adapted illustration of the skill Conditions of skill achievement	Obtaining a list of 12 items (after the initial proposal of 20 items)
Response process	Pilot study, search for error sources Adapting items Defining units of measurement	Interrater reproducibility Item content (redundant, inaccurate) Controlling the sources of measurement errors Weighing items	Fusion/removal of redundant items Minutes, degrees, centimeters justification
Internal structure	Internal coherence Reproducibility Discrimination of learners	Cronbach Alpha Coefficient, interrater: Cohen Kappa, ICC	Cronbach result Correlation between 2 raters
Comparison with other variables	Score vs success or failure of the procedure Score vs theoretical assessment Score vs previous experience/level of expertise	Correlation between procedure success or theoretical assessment and score with the tool	Time for success, score for success and rating
Consequences	Minimum passing score	Pass-fail score with procedure success	14/20

**Table 3:** Example of the development of an assessment tool for the observation of teamwork in simulation [48]

Source of validity	Method	Judgment criteria	Results
Content	1. Description of the *Clinical Teamwork Scale (CRM scale)* Development	Literature review Scale already used in another field (aeronautics)	15 items 5 categories 1 overall skill score
Response process	1. Relevance of items 2. weighting items 3. Raters’ training (moderate)	1. Precise description of each item 2. Quantitative criteria 3. Qualitative criteria 4. CRM principles	1. Ratings aid table 2. 0 to 10 or 0/1 Descriptive levels: not relevant/unacceptable/poor/average/good/perfect
Internal structure	1. Built-in validity 2. Scale usability 3. Reproducibility	1. Distribution of scores from the preset level 2. Number of items filled in full 3. interrater concordance, the correlation between overall score and categories (Kappa, Kendall, Pearsons, ICC) 4. Variance of each category	1. Score tailored to each level 2. Easy-to-use scale (little loss of information) 3. correlation between raters 4. Variation in scores between scenarios sources of error

A tool is validated in a language. Theoretically, this tool can only be used in this language, given the nuances present with interpretation [49]. In certain circumstances, a “translated” tool, but not a “translated and validated in a specific language” tool, can lead to semantic biases that can affect the meaning of the content and its representation [49–55]. For each assessment sequence, validity criteria consist of using different tools in different assessment situations and integrating them into a comprehensive program which considers all aspects of competency. The rating made with a validated tool for one situation must be combined with other assessment situations, since there is no “ideal” tool [28, 56] A given tool can be used with different professions or with participants at different levels of expertise or in different languages if it is validated for these situations [57, 58]. In a summative context, a tool must have demonstrated a high-level of validity to be used because of the high stake for the participants [56]. Finally, the use or creation of an assessment tool requires trainers to question its various aspects, from how it was created to its reproducibility and the meaning of the results generated [59, 60].

Two types of assessment tools should be distinguished: tools that can be adapted to different situations and tools that are specific to a situation [61]. Thus, technical skills may have a dedicated assessment tool (e.g., intraosseous) [47] or an assessment grid generated from a list of pre-established and validated items (e.g., TAPAS scale) [62]. Non-technical skills can be observed using scales that are not situation-specific (e.g., ANTS, NOTECHS) [63, 64] or that are situation-specific (e.g., TEAM scale for resuscitation) [57, 65]. Assessment tools should be provided to participants and should be included in the scenario framework, at least as a reference [66–69]. In the summative assessment of a procedure, structured assessment tools should probably be used, using a structured objective assessment form for technical skills [70]. The use of a scale, in the context of the assessment of a technical gesture, seems essential. As with other tools, any scale must be validated beforehand [47, 70–72].

#### Consequences of undergoing the simulation-based summative assessment process

Summative assessment has two notable consequences on learning strategies. First, it may drive the learner’s behavior during the assessment, while it is essential to assess the competencies targeted, not the ability of the participant to adapt to the assessment tool [6]. Second, the pedagogy key concept of “pedagogical alignment” must be respected [23, 73]. It means that assessment methods must be coherent with the pedagogical activities and objectives. For this to happen, participants must have formative simulation training focusing on the assessed competencies prior to the SBSA [24].

Participants have been reported as experiencing commonly mild (e.g., appearing slightly upset, distracted, teary-eyed, quiet, or resistant to participating in the debriefing) or moderate (e.g., crying, making loud, and frustrated comments) psychological events in the simulation [74]. While voluntary recruitment for formative simulation is commonplace, all students are required to take summative assessments in training. This required participation in high-stake assessment may have a more consequential psychological impact [26, 75]. This impact can be modulated by training and assessment conditions [75]. First, the repetition of formative simulations reduces the psychological impact of SBSA on participants [76]. Second, the transparency on the objectives and methods of assessment limits detrimental psychological impact [77, 78]. Finally, detrimental psychological impacts are increased by abnormally high physiological or emotional stress such as fatigue, and stressful events in the 36 h preceding the assessment, such that students with a history of post-traumatic stress disorder or psychological disorder may be strongly and negatively impacted by the simulation [76, 79–81].

It is necessary to optimize SBSA implementation to limit its pedagogical and psychological negative impacts. Ideally, during the summative assessment, it has been proposed to take into account the formative assessment that has already been carried out [1, 20, 21]. Similarly in continuing education, the professional context of the person assessed should be considered. In the event of failure, it will be necessary to ensure sympathetic feedback and to propose a new assessment if necessary [21].

#### Scenarios for simulation-based summative assessment

Some authors argue that there are differences between summative and formative assessment scenarios [76, 79–81]. The development of a SBSA scenario begins with the choice of a theme, which is most often agreed upon by experts at the local level [66]. The themes are most often chosen based on the participants’ competencies to be assessed and included in the competencies requirement for the initial [82] and continuing education [35, 83]. A literature review even suggests the need to choose themes covering all the competences to be assessed [41]. These choices of themes and objectives also depend on the simulation tools technically available: “The themes were chosen if and only if the simulation tools were capable of reproducing “a realistic simulation” of the case.” [84].

The main quality criterion for SBSA is that the cases selected and developed are guided by the assessment objectives [85]. It is necessary to be clear about the assessment objectives of each scenario to select the right assessment tool [86]. Scenarios should meet four main principles: predictability, programmability, standardizability, and reproducibility [25]. Scenario writing should include a specific script, cues, timing, and events to practice and assess the targeted competencies [87]. The implementation of variable scenarios remains a challenge [88]. Indeed, most authors develop only one scenario per topic and skill to be assessed [85]. There are no recommendations for setting a predictable duration for a scenario [89]. Based on our findings we suggest some key elements for structuring a SBSA scenario in Table 4. For technical skill assessment, scenarios will be short and the assessment is based on an analytical score [82, 89]. For non-technical skill assessment, scenarios will be longer and the assessment based on analytical and holistic scores [82, 89].

**Table 4:** Key element structuring a summative assessment scenario

Elements	Recommendations
Duration	10 to 15 min Short for technical skills Longer for non-technical skills
Objectives	Accurate list of competencies and skills to be assessed
Essential items	Initial assessment Diagnostic strategy Situation management Orientation strategy
Script	Computerized (programed if possible)
Rating scale	*Checklist, Global Rating Scale* Scale (20 to 30 items) Analytic score for technical skills Analytic and holistic (e.g., ANTS) for non-technical skills
Validation	Pilot sessions (scenario testing and rater training) 1 or 2 cases per student during scenario testing
Assessment	Video rating Cohen’s Kappa test for differences between raters Student’s *t* test for the ability to discriminate between students

#### Debriefing, video, and research for simulation-based summative assessment

Studies have shown that debriefings are essential in formative assessment [90, 91]. No such studies are available for summative assessment. Good practice requires debriefing in both formative and summative simulation-based assessments [92, 93]. In SBSA, debriefing is often brief feedback given at the end of the simulation session, in groups [85, 94, 95], or individually [83]. Debriefing can also be done later with a trainer and help of video, or via written reports [96]. These debriefings make it possible to assess clinical skills for summative assessment purposes [97]. Some tools have been developed to facilitate this assessment of clinical reasoning [97].

Video can be used for four purposes: session preparation, simulation improvement, debriefing, and rating (Table 5) [95, 98]. In SBSA sessions, video can be used during the prebriefing to provide participants with standardized and reproducible information [99]. A video can increase the realism of the situation during the simulation with ultrasound loops and laparoscopy footage. Simulation records can be reviewed either for debriefing or rating purposes [34, 71, 100, 101]. A video is very useful for the training raters (e.g., for calibration and recalibration) [102]. It enables raters to rate the participants’ performance offline and to have an external review if necessary [34, 71, 101]. Despite the technical difficulties to be considered [42, 103], it can be expected that video-based automated scoring assistance will facilitate assessments in the future.

**Table 5:** Uses of video for simulation-based formative and summative assessment

	**Formative assessment**	**Summative assessment**
**Prebriefing**	Participant information	
**Simulation**	Increased scenario realism (e.g., coelioscopy video)	
	Watching by observers	
**Immediate visualization after simulation**	Self-assessment	No self-assessment (in the literature)
	Debriefing by trainers (selected sequences)	
**Delayed visualization**	Learning teamwork or skills for a formative purpose	Deferred debriefing Rater training (calibration and recalibration) Administrative evidence

The constraints associated with the use of video rely on the participants’ agreement, the compliance with local rules, and that the structure in charge of the assessment with video secures the protection of the rights of individuals and data safety, both at a national and at the higher (e.g., European GDPR) level [104, 105].

In Table 5, we list the main uses of video during simulation sessions found in the literature.

Research in SBSA can focus, as in formative assessment, on the optimization of simulation processes (programs, structures, human resources). Research can also explore the development and validation of summative assessment tools, the automation and assistance of assessment resources, and the pedagogical and clinical consequences of SBSA.

#### Trainers for simulation-based summative assessment

Trainers for SBSA probably need specific skills because of the high number of potential errors or biases in SBSA, despite the quest for objectivity (Table 6) [106]. The difficulty in ensuring objectivity is likely the reason why the use of self or peer assessment in the context of SBSA is not well documented and the literature does not yet support it [59, 60, 107, 108].

**Table 6:** Potential errors, effects, and bias in simulation-based summative assessment [109, 110]

Type of error	Error description
Error of homogenization	Tendency to rate neither too good or too bad, making discrimination more difficult
Halo effect	Tendency to see everything right or wrong in the same performance
Time effect	Bias related to observations of early or late good or bad performance during sessions
Bias of “clemency”	Willingness not to give bad grades
Repository error	Judgment based on what the rater would have done and not on the assessment tool
Group effect	Evaluation based on the team’s performance rather than the participant’s performance

SBSA requires the development of specific scenarios, staged in a reproducible way, and the mastery of assessment tools to avoid assessment bias [111–114]. Fulfilling those requirements calls for specific abilities to fit with the different roles of the trainer. These different roles of trainers would require specific initial and ongoing training tailored to their tasks [111, 113]. In the future, concepts of the roles and tasks of these trainers should be integrated into any “training of trainers” in simulation.

#### Implementation of simulation-based summative assessment in healthcare

The use of SBSA has been described by Harden in 1975 with Objective and Structured Clinical Examination (OSCE) tests for medical students [115]. The summative use of simulation has been introduced in different ways depending on the professional field and the country [116]. There is more literature on certification at the undergraduate and graduate levels than on recertification at the postgraduate level. The use of SBSA in re-certification is currently more limited [83, 117]. Participation is often mandated, and it does not provide a formal assessment of competency [83]. Some countries are defining processes for the maintenance of certification in which simulation is likely to play a role (e.g., in the USA [118] and France [116]). Recommendations regarding the development of SBSA for OSCE were issued by the AMEE (Association for Medical Education in Europe) in 2013 [12, 119]. Combined with other recommendations that address the organization of examinations on other immersive simulation modalities, in particular, full-scale sessions using complex mannequins [22, 85], they give us a solid foundation for the implementation of SBSA.

The overall process to ensure a high-quality examination by simulation is therefore defined but particularly demanding. It mobilizes many material and human resources (administrative staff, trainers, standardized patients, and healthcare professionals) and requires a long development time (several months to years depending on the stakes) [36]. We believe that the steps to overcome during the implementation of SBSA range from setting up a coordination team, to supervising the writers, the raters, and the standardized patients, as well as taking into account the logistical and practical pitfalls.

The development of a competency framework valid for an entire curriculum (e.g., medical studies) satisfies a fundamental need [7, 120]. This development allows identifying competencies to be assessed with simulation, those to be assessed by other methods, and those requiring triangulation by several assessment methods. This identification then guides scenarios’ writing and examination’s development with good content validity. Scenarios and examinations will form a bank of reproducible assessment exercises. The examination quality process, including psychometric analyses, is part of the development process from the beginning [85].

We have summarized in Table 7 the different steps in the implementation of SBSA.

**Table 7:** Implementation of simulation-based summative assessment step by step

Items	Goals	Modalities
Team	Identify the training staff	Structure coordination Size the team: skills, time available, stability (project over several months/years)
Competencies repository	Create the competencies repository to be assessed	Expert panels Define the number and type of examination needed Must be known to students
Curriculum	integrate summative assessment in the curriculum	Pedagogical alignment: summative part drives the formative part of the curriculum No summative assessment without pre-simulation exposure Intermediate summative assessment could be useful [121]
Examination	Define summative assessment modalities through simulation	Length and number of scenarios stations [122, 123] The higher the fidelity of the examination, the harder is it to set it up, the lower the feasibility
Scenarios	Develop a bank of scenarios and rating grids [124]	Choose the editors for the scenarios Write the scenarios Scenarios’ peer-review and test Establish/choose assessment tools (Checklist or global scale) Set the minimum passing score The themes of the bank's scenarios cover the competencies of the repository
Training raters	Limit rating variations for a given performance	Choice of raters Raters’ Training Workshop
Standardized Patients	Develop a standardized patient pool	Recruitment, selection, training, and standardization [125]
D-Day	How the examination take place	Logistics: e.g., dates, rooms, standardized patients, rights of personal portrayal, GDPR Participants’ path, breaks Materials to supply, to be brought by students (e.g., stethoscope) Examination-adapted briefings Problems to anticipate: e.g., maintenance of standardization, failure or breakage of equipment, backup paper supports, dedicated staff for support to stressed participants,
immediately after examination	Finalize the examination	Collect and check assessment grids for early detection of inconsistencies, rating oversights, missing data Management of participants’ complaints and plea
Quality process	Prepare future examination	Identify potential changes to do to some scenarios Removal of inappropriate scenarios: e.g., too long, misleading, source of rating inconsistency, Changes to standardized patients’ training Changes in raters’ training

##### Recertification

Recertification programs for various healthcare domains are currently being implemented or planned in many countries (e.g., in the USA [118] and France [116]). This is a continuation of the movement to promote the maintenance of competencies. Examples can be cited in France with the creation of an agency for continuing professional development or in the USA with the Maintenance Of Certification [83, 126]. The certification of health care facilities and even teams is also being studied [116]. Simulation is regularly integrated into these processes (e.g., in the USA [118] and France [116]). Although we found some commonalities basis between the certification and recertification processes, there are many differences (Table 8).

**Table 8:** Commonalities and discrepancies between certification and recertification

Items	Commonalities	Discrepancies
Modalities	Multimodal process (course, simulation, etc.) [34, 83, 92] Field follow-up opportunities [35]	Low percentage of existing recertification [34, 83] Level of acceptability and feasibility of recertification Level of recertification: pursuing individual certification or switching with team recertification
Organization bodies	Accredited centers (functional specification) [34, 83] Same rigor in setting up	Can institutions (universities, schools) in charge of certification, provide recertification?
Objectives	Targeted level of competency	Difficulties in the efficient selection of competencies to be assessed with recertification: ^•^Multiple constraints (time/means) ^•^Communication/teamwork, performance gaps, frequent adverse events? Scenarios and assessment tools adapted for learning objectives [127]
Consequences	Possible failure of certification or recertification	The impact of a failure to recertification is major for a professional Mandatory discretion of the recertification process Opportunity for screening of professionals in difficulty (burn out…) [92, 116]
Funding	Funding difficulties	Many options of financing in recertification (state, professional insurance, etc.)

Currently, when simulation-based training is mandatory (e.g., within the American Board of Anesthesiology’s “Maintenance Of Certification in Anesthesiology,” or MOCA 2.0® in the US), it is most often a formative process [34, 83]. SBSA has a place in the recertification process, but there are many pitfalls to avoid. In the short term, we believe that it will be easier to incorporate formative sessions as a first step. The current consensus seems to be that there should be no pass/fail recertification simulation without personalized global professional support, but which is not limited to a binary aptitude/inaptitude approach [21, 116].

## Discussion

Many important issues and questions remain regarding the field of SBSA. This discussion will return to our identified 7 topics and highlight these points, their implications for the future, and some possible leads for future research and guidelines development for the safe and effective use of this tool in SBSA.

### What can be assessed in simulation?

SBSA is currently mainly used in initial training in uni-professional and individual settings via standardized patients or task trainers (OSCE) [12, 13]. In the future, SBSA will also be used in continuing education for professionals who will be assessed throughout their career (re-certification) as well as in interprofessional settings [83]. When certifying competencies, it is important to keep in mind the differences between the desired competencies and the observed performances [128]. Indeed, it must be that “what is a competency” is specifically defined [6, 19, 21]. Competencies are what we wish to evaluate during the summative assessment to validate or revalidate a professional for his/her practice. Performance is what can be observed during an assessment [20, 21]. In this context, we consider three unresolved issues. The first issue is that an assessment only gives access to a performance at a given moment (“Performance is a snapshot of a competency”), whereas one would like to assess a competency more generally [128]. The second issue is: How does an observed performance—especially in simulation—reveal a real competency in real life? [129] In other words, does the success or failure of a single SBSA really reflect actual real-life competency? [130] The third issue is the assessment of a team performance/competency [131–133]. Until now, SBSA has come from the academic field and has been an individual assessment (e.g., OSCE). Future SBSA could involve teams, driven by governing bodies, institutions, or insurances [134, 135]. The competency of a team is not the sum of the competencies of the individuals who compose it. How can we proceed to assess teams as a specific entity, both composed of individuals and independent of them? To make progress in answering these three issues, we believe it is probably necessary to consider the approximation between observed and assessed performance and competency as acceptable, but only by specifying the scope of validity. Research in these areas is needed to define it and answer these questions.

The consequence of undergoing SBSA has focused on the psychological aspect and have set aside the more usual consequences such as achieving (or not) the minimum passing score. Future research should embrace more global SBSA consequence field, including how reliable SBSA is at determining how someone is competent.

### Assessment tools for simulation-based summative assessment

Rigor and method in the development and selection of assessment tools are paramount to the quality of the summative assessment [136]. The literature shows that is necessary that assessment tools be specific to their intended use that their intrinsic characteristics be described and that they be validated [38, 40, 41, 137]. These specific characteristics must be respected to avoid two common issues [1, 6]. The first issue is that of a poorly designed or constructed assessment tool. This tool can only give poor assessments because it will be unable to capture performance correctly and therefore to approach the skill to be assessed in a satisfactory way [56]. The second issue is related to poor or incomplete tool evaluation or inadequate tool selection. If the tool is poorly evaluated, its quality is unknown [56]. The scope of the assessment that is done with it is limited by the imprecision of the tool’s quality. If the tool is poorly selected, it will not accurately capture the performance being assessed. Again, summative assessment will be compromised. It is currently difficult to find tools that meet all the required quality and validation criteria [56]. On the one hand, this requires complex and rigorous work; on the other hand, the potential number of tools required is large. Thus, the overall volume of work to rigorously produce assessment tools is substantial. However, the literature provides the characteristics of validity (content, response process, internal structure, comparison with other variables, and consequences), and the process of developing qualitative and reliable assessment tools [38–41, 45]. It therefore seems important to systematize the use of these guidelines for the selection, development, and validation of assessment tools [137]. Work in this area is needed and network collaboration could be a solution to move forward more quickly toward a bank of valid and validated assessment tools [39].

### Consequences of undergoing the simulation-based summative assessment process

We had focused our discussion on the consequences of SBSA excluding the determining of the competencies and passing rates. Establishing and maintaining psychological safety is mandatory in simulation [138]. Considering the psychological and physiological consequences of SBSA is fundamental to control and limit negative impacts. Summative assessment has consequences for both the participants and the trainers [139]. These consequences are often ignored or underestimated. However, these consequences can have an impact on the conduct or results of the summative assessment. The consequences can be positive or negative. The “testing effect” can have a positive impact on long-term memory [139]. On the other hand, negative psychological (e.g., stress or post-traumatic stress disease), and physiological (e.g., sleep) consequences can occur or degrade a fragile state [139, 140]. These negative consequences can lead to questioning the tools used and the assessments made. These consequences must therefore be logically considered when designing and conducting the SBSA. We believe that strategies to mitigate their impact must be put in place. The trainers and the participants must be aware of these difficulties to better anticipate them. It is a real duality for the trainer: he/she has to carry out the assessment in order to determine a mark and at the same time guarantee the psychological safety of the participants. It seems fundamental to us that trainers master all aspects of SBSA as well as the concept of the safe container [138] to maximize the chances of a good experience for all. We believe that ensuring a fluid pedagogical continuum, from training to (re)certification in both initial and continuing education using modern pedagogical techniques (e.g., mastery learning, rapid cycle deliberate practice) [141–144] could help maximize the psychological and physiological safety of participants.

### Scenarios for simulation-based summative assessment

The structure and use of scenarios in a summative setting are unique and therefore require specific development and skills [83, 88]. SBSA scenarios differ from formative assessment scenarios by the different educational objectives that guide their development. Summative scenarios are designed to assess a skill through observation of performance, while formative scenarios are designed to learn and progress in mastering this same skill. Although there may be a continuum between the two, they remain distinct. SBSA scenarios must be predictable, programmable, standardizable, and reproductible [25] to ensure fairly assessed performances among participants. This is even more crucial when standardized patients are involved (e.g., OSCE) [119, 145]. In this case, a specific script with expectations and training is needed for the standardized patient. The problem is that currently there are many formative scenarios but few summative scenarios. The rigor and expertise required to develop them is time-consuming and requires expert trainer resources. We believe that a goal should be to homogenize the scenarios, along with preparing the human resources who will implement them (trainers and standardized patients) and their application. We believe one solution would be to develop a methodology for converting formative scenarios into summative ones in order to create a structuring model for summative scenarios. This would reinvest the time and expertise already used for developing = formative scenarios.

### Debriefing for simulation-based summative assessment

The place of debriefing in SBSA is currently undefined and raises important questions that need exploration [77, 90, 146–148]. Debriefing for formative assessment promotes knowledge retention and helps to anchor good behaviors while correcting less ideal ones [149–151]. In general, taking an exam promotes learning of the topic [139, 152]. Formative assessment without a debriefing has been shown to be detrimental, so it is reasonable to assume that the same is true in summative assessment [91]. The ideal modalities for debriefing in SBSA are currently unknown [77, 90, 146–148]. Integrating debriefing into SBSA raises a number of organizational, pedagogical, cognitive, and ethical issues that need to be clarified. From an organizational perspective, we consider that debriefing is time and human resource-consuming. The extent of the organizational impact varies according to whether the feedback is automatized, standardized, personalized, and collective or individual. From an educational perspective, debriefing ensures pedagogical continuity and continued learning. We believe this notion is nuanced, depending on whether the debriefing is integrated into the summative assessment or instead follows the assessment while focusing on formative assessment elements. We believe that if the debriefing is part of the SBSA, it is no longer a “teaching moment.” This must be factored into the instructional strategy. How should the trainer prioritize debriefing points between those established in advance for the summative assessment and those that would emerge from any individuals’ performance? From a cognitive perspective, whether the debriefing is integrated into the summative assessment may alter the interactions between the trainer and the participants. We believe that if the debriefing is integrated into the SBSA, the participant will sometimes be faced with the cognitive dilemma of whether to express his/her “true” opinions or instead attempt to provide the expected answers. The trainer then becomes uncertain of what he/she is actually assessing. Finally, from an ethical perspective, in the case of a mediocre or substandard clinical performance, there is a question of how the trainer resolves discrepancies between observed behavior and what the participant reveals during the debriefing. What weight should be given to the simulation and to the debriefing for the final rating? We believe there is probably no single solution to how and when the debriefing is conducted during a summative assessment but rather promote the idea of adapting debriefing approaches (e.g., group or individualized debriefing) to various conditions (e.g., success or failure in the summative assessment). These questions need to be explored to provide answers as to how debriefing should be ideally conducted in SBSA. We believe a balance must be found that is ethically and pedagogically satisfactory, does not induce a cognitive dilemma for the trainer, and is practically manageable.

### Trainers for simulation-based summative assessment

The skills and training of trainers required for SBSA are crucial and must be defined [136, 153]. We consider that skills and training for SBSA closely mirror skills and training needed for formative assessment in simulation. This continuity is part of the pedagogical alignment. These different steps have common characteristics (e.g., rules in simulation, scenario flow) and specific ones (e.g., using assessment tools, validating competence). To ensure pedagogical continuity, the trainers who supervise these courses must be trained in and be masterful in simulation, adhering to pedagogical theories. We believe training for SBSA represents new skills and a potentially greater cognitive load for the trainers. It is necessary to provide solutions to both of these issues. For the new skills of trainers, we consider it necessary to adapt or complete the training of trainers by integrating knowledge and skills needed for properly conducting SBSA: good assessment practices, assessment bias mitigation, rater calibration, mastery of assessment tools, etc. [154]. To optimize the cognitive load induced by the tasks and challenges of SBSA, we suggest that it could be helpful to divide the tasks between the different trainers’ roles. We believe that conducting a SBSA therefore requires three types of trainers whose training is adapted to their specific role. First, three are the trainer-designers who are responsible for designing the assessment situation, selecting the assessment tool(s), training the trainer-rater(s), and supervising the SBSA sessions. Second, there should be the trainer-operators responsible for running the simulation conditions that support the assessment. Third, there are the trainer-raters who conduct the assessment using the assessment tool(s) selected by the trainer-designer(s) for which these trainer-raters have been specifically trained. The high-stake nature of SBSA requires a high level of rigor and professionalism from the three levels of trainers, which implies they have a working definition of the skills and the necessary training to be up to the task.

### Implementing simulation-based summative assessment in healthcare

Implementing SBSA is delicate, requires rigor, respect for each step, and must be evidence-based. While OSCEs are simulation-based, simulation is not limited to OSCEs. Summative assessment with OSCEs has been used and studied for many years [12, 13]. This literature is therefore a valuable source for learning lessons about summative assessment applied to simulation as a whole [22, 85, 155]. Knowledge from OSCE summative assessment needs to be supplemented so that simulation can perform summative assessment according to good evidence-based practices. Given the high stakes of SBSA, we believe it necessary to rigorously and methodically adapt what is already validated during implementation (e.g., scenarios, tools) and to proceed with caution for what has not yet been validated. As described above, many steps and prerequisites are necessary for optimal implementation, including (but not limited to) identifying objectives; identifying and validating assessment tools; preparing simulations scenarios, trainers, and raters; and planning a global strategy beginning from integrating the summative assessment in the curriculum to the managing the consequences of this assessment. SBSA must be conducted within a strict framework for its own sake and that of the people involved. Poor implementation would be detrimental to all participants, trainers, and the practice SBSA. This risk is greater for recertification than for certification [156], while initial training is able to accommodate SBSA easily because it is familiar (e.g., trainees engage in OSCEs at some point in their education), including SBSA in recertifying practicing professionals is not as obvious and may be context-dependent [157]. We understand that the consequences of failed recertification are potentially more impactful, both psychologically and for professional practice. We believe that solutions must be developed, tested, and validated that both fill gaps and preserve professionals and patients. Implementing SBSA therefore must be progressive, rigorous, and evidence-based to be accepted and successful [158].

### Limitations

This work has some limitations that should be emphasized. First, this work covers only a limited number of issues related to SBSA. The entire topic is possibly not covered and we may not have explored other questions of interest. Nevertheless, the NGT methodology allowed this work to focus on those issues that were most relevant and challenging to the panel. Second, the literature review method (state-of-the-art literature reviews expanded with a snowball technique) does not guarantee exhaustiveness, and publications on the topic may have escaped the screening phase. However, it is likely that we have identified key articles focused on the questions explored. Potentially unidentified articles would therefore either not be important to the topic or would address questions not selected by the NGT. Third, this work was done by a French-speaking group, and a Francophone-specific approach to simulation, although not described to our knowledge, cannot be ruled out. This risk is reduced by the fact that the work is based on international literature from different specialties in different countries and that the panelists and reviewers were from different countries. Fourth, the analysis and discussion of the consequences of SBSA were focused on psychological consequences. This does not cover the full range of consequences including the impact on subsequent curricula or career pathways. Data in the literature exist on the subject and probably deserve a specific body of work. Despite these limitations, however, we believe this work is valuable because it raises questions and offers some leads toward solutions.

## Conclusions

The use of SBSA is very promising with a growing demand for its application. Indeed, SBSA is a logical extension of simulation-based formative assessment and competency-based medical education development. It is probably wise to anticipate and plan for approaches to SBSA, as many important moving parts, questions, and consequences are emerging. Clearly identifying these elements and their interactions will aid in developing reliable, accurate, and reproducible models. All this requires a meticulous and rigorous approach to preparation commensurate with the challenges of certifying or recertifying the skills of healthcare professionals. We have explored the current knowledge on SBSA and have now shared an initial mapping of the topic. Among the seven topics investigate, we have identified (i) areas with robust evidence (what can be assessed with simulation?); (ii) areas with limited evidence that can be assisted by expert opinion and research (assessment tools, scenarios, and implementation); and (iii) areas with weak or emerging evidence requiring guidance by expert opinion and research (consequences, debriefing, and trainers) (Fig. 1). We modestly hope that this work can help reflection on SBSA for future investigations and can drive guideline development for SBSA.

## Data Availability

All data generated or analyzed during this study are included in this published article.

## Abbreviations

GDPR: General data protection regulation
NGT: Nominal group technique
OSCE: Objective structured clinical examination
SBSA: Simulation-based summative assessment
